# Validation of accelerometry as a digital phenotyping measure of negative symptoms in schizophrenia

**DOI:** 10.1038/s41537-022-00241-z

**Published:** 2022-04-15

**Authors:** Gregory P. Strauss, Ian M. Raugh, Luyu Zhang, Lauren Luther, Hannah C. Chapman, Daniel N. Allen, Brian Kirkpatrick, Alex S. Cohen

**Affiliations:** 1 Department of Psychology, University of Georgia, Athens, GA USA; 2 Department of Psychology, University of Nevada, Las Vegas, NV USA; 3 Department of Psychiatry and Behavioral Sciences, University of Nevada, Reno School of Medicine, Las Vegas, NV USA; 4 Department of Psychology, Louisiana State University, Baton Rouge, LA USA

**Keywords:** Schizophrenia, Psychosis

## Abstract

Negative symptoms are commonly assessed via clinical rating scales; however, these measures have several inherent limitations that impact validity and utility for their use in clinical trials. Objective digital phenotyping measures that overcome some of these limitations are now available. The current study evaluated the validity of accelerometry (ACL), a passive digital phenotyping method that involves collecting data on the presence, vigor, and variability of movement. Outpatients with schizophrenia (SZ: *n* = 50) and demographically matched healthy controls (CN: *n* = 70) had ACL continuously recorded from a smartphone and smartband for 6 days. Active digital phenotyping assessments, including surveys related to activity context, were also collected via 8 daily surveys throughout the 6 day period. SZ participants had lower scores on phone ACL variables reflecting vigor and variability of movement compared to CN. ACL variables demonstrated convergent validity as indicated by significant correlations with active digital phenotyping self-reports of time spent in goal-directed activities and clinical ratings of negative symptoms. The discriminant validity of ACL was demonstrated by low correlations with clinical rating scale measures of positive, disorganized, and total symptoms. Collectively, findings suggest that ACL is a valid objective measure of negative symptoms that may complement traditional approaches to assessing the construct using clinical rating scales.

## Introduction

Negative symptoms significantly limit potential for successful functional outcome and recovery in individuals with schizophrenia^1–3^. Unfortunately, currently available psychosocial and pharmacological treatments yield minimal benefits for negative symptoms^4^ and no drug has received United States Food and Drug Administration (FDA) approval for an indication of negative symptoms.

In an effort to make progress in the treatment of negative symptoms, the United States National Institute of Mental Health (NIMH) hosted a consensus development conference in 2005. Several key conclusions resulted from this meeting. Among these were that: 1) at least 5 core domains exist within the negative symptom construct (blunted affect, alogia, avolition, anhedonia, asociality); and 2) first-generation clinical rating scales (e.g., SANS, BPRS, PANSS, NSA) were inadequate for measuring the construct, and new assessment tools were needed to evaluate the 5 consensus domains according to modern conceptualizations^5^.

Following the Consensus Development conference, a workgroup was formed to create a clinical rating scale that would meet these needs in the field. In the process, it became clear that two instruments were needed: one that would address the essential elements of each domain but be concise enough to be practical for routine clinical use as well as large multicenter clinical trials and another that would cover each of the domains in extensive detail. As a consequence, the workgroup divided into 2 groups to accomplish these goals and two second-generation rating instruments were developed: the Brief Negative Symptom Scale (BNSS)^6^ and Clinical Assessment Interview for Negative Symptoms (CAINS)^7^. Excellent psychometric properties have been demonstrated for the original English versions of the BNSS^6,8–13^ and the CAINS^7,14,15^, as well as translated versions of both scales^16–32^. Although there is considerable overlap in how the BNSS and CAINS assess the 5 domains identified in the consensus conference, they each have important practical and conceptual differences that offer distinct advantages for use in clinical trials and laboratory-based experimental psychopathology studies^11^.

Although second-generation scales, such as the BNSS and CAINS, represent an important advance from first-generation scales, they are subject to several limitations that are inherent to all clinical interview-based measures. For example, clinical ratings are: (1) influenced by cognitive impairments (e.g., long-term and working memory impairments) that make retrospective and prospective reports less accurate^33^; (2) subject to biases and self-report confounds, such as social desirability, over and under reporting tendencies, halo effects, and biases resulting from patient/interviewer characteristics (e.g., gender, ethnicity); (3) impacted by limited precision and resolution (e.g., requiring raters to average across distinct contexts and lengthy time intervals that may hold meaningful variance). Clinical rating scales are also costly in terms of financial and time costs needed to implement them in clinical trials, and they may not be highly sensitive to treatment effects.

It may now be possible to circumvent these limitations associated with clinical rating scales using what may become the “third generation” of negative symptom assessment: digital phenotyping. Digital phenotyping refers to the use of mobile devices (e.g., wearable smartbands, smart phones) to initiate the collection of data in everyday life^34,35^. A distinction is made between digital phenotyping approaches that are passive versus active. Passive digital phenotyping involves the unobtrusive collection of objective data, typically from the internal sensors of a device (e.g., smartphone or band). Several types of passive digital phenotyping variables can be collected that may hold relevance to negative symptoms, such as phone usage data (e.g., call/text logs, screen time, Bluetooth connectivity), social media data (e.g., time spent on apps like Facebook or Instagram), geolocation (GPS coordinate data), speech samples collected from ambient sound in the environment, and ambulatory psychophysiology. These objective, passive digital phenotyping variables can be paired with active digital phenotyping methods that require the participant to choose to perform an activity or behavior of interest (e.g., a survey, video, or cognitive task on the phone). When passive data is scaled and epoched to the same time interval as the active data, the combined approach allows for a more nuanced examination of symptoms as they unfold in real life (e.g., when the participant is in a certain mood state, engaged in a social interaction, or in a certain location)^36^. To date, few studies have evaluated the utility of combining active and passive digital phenotyping measures to evaluate negative symptoms in SZ.

The aim of the current study was to provide an initial psychometric evaluation of one promising passive digital phenotyping measure of negative symptoms: accelerometry (ACL). ACL involves calculating the presence, vigor, and variability of motor movement. ACL is similar to other measures of motor behavior in real life, such as actigraphy; however, unlike these other measures, which rely on specialized sensors/instruments designed for research purposes, ACL can be collected via the internal sensors of smartphones or smartbands that are commercially available and thus more feasible for routine use. Accelerometers have received approval as medical devices from the U.S. Food and Drug Administration for a number of uses related to daily activity, falls, and sleep. They have also been used as outcome measures in clinical trials measuring physical and mental health outcomes^37^. When paired with active digital phenotyping approaches (e.g., surveys), ACL has the potential to lead to a more granular understanding of human activity across contexts (e.g., locations, activity types, social settings), including an understanding of goal-directed behaviors encapsulated in the negative symptom construct.

Few studies have explicitly evaluated accelerometry as a method for assessing symptoms of SZ. In an epidemiological study in the United Kingdom, Firth et al.^38^ evaluated the utility of ACL for objectively evaluating physical activity relative to self-reported physical activity; although individuals with SZ had no self-reported reductions in physical activity, ACL indicated an objective reduction in amount of movement. Ben-Zeev and colleagues have used ACL in their Cross-Check symptom prediction and relapse monitoring platform, demonstrating that various ACL measures predict relapse and symptom severity on a 7-item version of the Brief Psychiatric Rating Scale^39–41^; however, given that the BPRS does not measure the volitional component of negative symptoms^42^ and that convergence with aggregate BPRS scores was evaluated, it is unclear whether ACL metrics are valid measures of negative symptoms specifically.

To our knowledge, no study has directly explored the validity of ACL as a measure of negative symptoms in the context of everyday life, while pairing ACL with active digital phenotyping data to evaluate validity. We asked participants to perform 6 days of data collection, where active surveys evaluating symptoms and context (location, activity, social interaction) were sent 8 times per day and ACL data was collected continuously via smartphone and a wrist-worn smartband. The following hypotheses were made: (1) SZ would have lower scores than CN on ACL mean and standard deviation variables measuring the presence/vigor of movement and variability of movement; (2) convergent validity of ACL would be demonstrated with: concurrently collected active self-reports of goal-directed activity context, as well as clinically rated negative symptoms measured via standard clinical interviews; (3) discriminant validity would be supported by low and nonsignificant correlations with measures of positive, disorganized, and total psychiatric symptoms collected via standard clinical rating scale measures.

## Results

### Hypothesis 1: Group differences in accelerometry variables

**Fig. 1:**
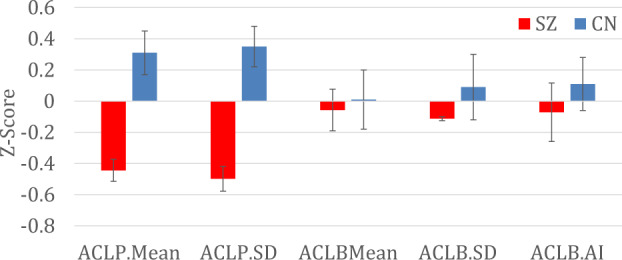
Group comparisons for accelerometry measured via smartphone and smartband. SZ schizophrenia, CN control, ACLP.Mean accelerometry phone mean, ACLP.SD accelerometry phone average standard deviation, ACLB.Mean accelerometry band mean, ACLB.AI accelerometry band activity index. Error bars represent standard errors.

SZ had significantly lower scores than CN for phone ACL mean, *F* (1, 118) = 17.75, *p* < 0.001, and ACL SD, *F* (1, 118) = 23.64, *p* < 0.001. Groups did not significantly differ on band ACL mean, *F* (1, 64) = 0.06, *p* = 0.80, ACL SD, *F* (1, 64) = 0.60, *p* = 0.44, or ACL Activity Index, *F* (1, 64) = 0.47, *p* = 0.50. Group comparisons are presented as Z-scores in Fig. 1 to facilitate ease of comparison across different units of measurement.

Exploratory analyses were also conducted for each of the phone and band ACL variables based on two types of contexts that should in theory demonstrate differences when participants are in that context versus not: public location context and recreational activity context (see Supplementary Table S1 in Supplemental Materials). Phone ACL showed significant or trend level higher mean and SD when participants in both groups reported being in public and while engaged in a recreational activity compared to when they were not in these contexts. Band measures did not demonstrate differences across contexts. These findings support the validity of the phone ACL metrics by indicating greater movement during real-world contexts where greater magnitude and variability of movement would be expected.

### Hypothesis 2: Convergent validity

Convergent validity was demonstrated within the SZ sample via significant negative correlations between band accelerometry mean and SD measures and clinically rated negative symptoms on the BNSS. Phone ACL metrics were not significantly associated with negative symptoms on the BNSS (see Table 1).

**Table 1:** Convergent validity correlations.

	ACLP.mean	ACLP.SD	ACLB.Mean	ACLB.SD	ACLB.AI
BNSS anhedonia	−0.12	−0.21	−0.45*	0.28	0.33
BNSS asociality	0.09	−0.09	−0.34	0.11	0.33
BNSS avolition	0.16	−0.14	−0.41*	0.39*	0.38
BNSS blunted affect	−0.13	−0.18	−0.40*	0.36	0.27
BNSS alogia	0.17	0.02	−0.40*	0.27	0.26

*ACLP.Mean* accelerometry phone mean, *ACLP.SD* accelerometry phone average standard deviation, *ACLB.Mean* accelerometry band mean, *ACLB.SD* accelerometry phone average standard deviation, *ACLB.AI* accelerometry band activity index, *BNSS* Brief Negative Symptom Scale.

Bold values indecate statistical significance *p* < 0.05; *p < 0.05; **p < 0.01; ***p < 0.001.

The EMA data also supported convergent validity of ACL. The proportion of time spent in goal-directed activities was significantly correlated with phone ACL mean (*r* = 0.45, *p* < 0.001) and SD (*r* = 0.39, *p* < 0.001) variables, such that greater vigor and variability in movement were associated with more goal-directed activity across the entire sample of SZ and CN participants. Band ACL metrics were not significantly correlated with EMA reported goal-directed activity (*r*’s < 0.09).

We also explored the association between band and phone ACL scores. No associations were significant in schizophrenia (*r*’s < 0.23) or controls (*r*’s < 0.10) (see Supplementary Table S2 in Supplemental Materials for complete correlations).

### Hypothesis 3: Discriminant validity

Discriminant validity was supported by low and nonsignificant correlations between ACL phone and band measures and clinically rated positive, disorganized, and total symptoms on the PANSS (see Table 2). Additionally, the higher magnitude nonsignificant correlations were in the opposite direction of what would be expected to indicate poor discriminant validity (i.e., greater positive and general symptoms associated with greater movement).

**Table 2:** Discriminant validity correlations.

	ACLP.mean	ACLP.SD	ACLB.Mean	ACLB.SD	ACLB.AI
PANSS positive	0.25	0.14	0.00	0.12	0.01
PANSS disorganized	−0.01	0.24	−0.08	0.17	−0.17
PANSS total	0.12	0.27	−0.24	0.36	0.22

*ACLP.Mean* accelerometry phone mean, *ACLP.SD* accelerometry phone average standard deviation, *ACLB.Mean* accelerometry band mean, *ACLB.AI* accelerometry band activity index, *PANSS* Positive and Negative Syndrome Scale.

*p < 0.05; **p < 0.01; ***p < 0.001.

## Discussion

The current study evaluated the validity of accelerometry as a passive digital phenotyping measure of negative symptoms in outpatients with SZ. Results generally supported the validity of accelerometry, as indicated by: (1) Criterion Validity: lower mean and SD phone ACL scores in SZ than CN; (2) Criterion Validity: Greater mean and SD phone ACL scores during contexts where participants reported being in public and engaged in recreational activities; (3) Convergent Validity: significant inverse correlations between band ACL measures and clinically rated negative symptoms on the BNSS; (4) Convergent Validity: significant positive correlations between phone ACL measures and EMA self-reports of engagement in goal-directed activity; (5) Discriminant Validity: nonsignificant and low correlations between ACL phone and band measures and positive, disorganized, and total symptoms on the PANSS.

Collectively, these findings provide proof-of-concept that ACL is feasible for use as an objective measure of negative symptoms in research studies. We recommend the following when incorporating ACL into research studies on negative symptoms: (1) Collect both band and phone ACL data, as different patterns of group differences and correlations emerged across these devices. Both methods of collection demonstrated some validity, and it is currently unclear why they diverged and had limited overlap. Our sample had difficulty pairing the band and phone, which resulted in some loss of band data; we suspect this was device specific and believe alternate bands may prove more feasible for participants to troubleshoot and implement consistently (see Raugh et al.^43^) for a discussion of adherence of passive digital phenotyping); (2) Band and phone ACL data both require intensive data processing; to be implemented in clinical trials, pharmaceutical companies may want to partner with experts in academia. Care should be taken with regard to cleaning and data reduction for phone versus band data, as these require different approaches due to the effects of gravitational forces and other collection characteristics; (3) Devices used to collect ACL, apps, and metrics used for scoring should undergo extensive validation processes before being implemented in clinical trials as outcome measures. The 3 V approach proposed by Goldsack et al.^44^ represents one model for doing so; (4) Evaluate a range of ACL metrics that reflect both vigor of movement and variability of movement; (5) The combination of passive ACL data and EMA-based surveys (e.g., context reports related to goal-directed activity) may yield more comprehensive estimates of negative symptoms than either active or passive digital phenotyping methods alone. Similarly, the EMA survey data may allow researchers to hone in on specific real-world contexts where passive digital phenotyping might be most relevant (e.g., when participants report being in public or engaged in goal-directed activities).

Certain limitations should be considered. First, this study was designed as proof-of-concept. More extensive investigations are needed with larger samples. This is particularly true for band-based ACL, as a portion of our sample had missing data due to bluetooth pairing problems. It is unclear if the participants who were not able to produce band data are comparable to those who were able to troubleshoot the band and produce data for both devices. Future studies should continue to compare band and phone data. We suspect the observed discrepancies may occur for a variety of reasons (e.g., participants may not carry the phone on them continuously, differences in how the accelerometers register movement). Second, participants used smartphones and bands that were provided to them for the study. It is unclear whether different patterns of data might emerge when participants use their own devices, and additional work is needed to confirm that ACL data is comparable across different operating systems and phones. Third, participants received payment for completing surveys and a bonus for returning the smart phone and band; it is unclear whether payment affected data acquisition, adherence, or quality. Fourth, data was collected for only 6 days. It is possible that longer periods of data collection could produce different results. Future studies should examine this issue directly, as it holds relevance for clinical trials. Further, although participants were instructed to carry the study phone and wear the band, participants may have not always been able to adhere to these procedures; this time period would not adequately capture a participant’s ACL. Finally, correlation coefficients were in the medium range, and lower than what might be expected to demonstrate strong convergent validity. Temporally proximal EMA measures tended to have better convergent validity than retrospective clinical rating scales with the passive ACL measures. This discrepancy likely reflects temporal resolution, precision of measurement, and/or methods variance rather than inadequate convergent validity.

Despite these limitations, findings suggest that markers of movement vigor and variability, such as those measured via accelerometry or actigraphy^45–47^, hold promise as objective passive digital phenotyping measures of negative symptoms in SZ. Using this same sample, we previously demonstrated that SZ participants report high tolerability when completing active and passive digital phenotyping. However, feasibility and adherence differed based on device. In particular, adherence (the % data received that was expected) was much lower for ACL measured via band (20%) than phone (87%) in SZ^43^. Similarly, feasibility of the band was also poorer than the phone, with SZ participants reporting difficulties pairing the band and phone via bluetooth, whereas phone ACL demonstrated minimal feasibility issues (e.g., remembering to carry the phone). When coupled with the validity findings reported above, our prior tolerability/adherence/feasibility findings^43^ suggest that while ACL may hold promise, methodological considerations are warranted. Until further validity is demonstrated and it is determined whether band or phone collection (or both in tandem) is most ideal, clinical trials may want to use ACL as a secondary or exploratory outcome measure. We believe it could be particularly useful when added to multi-level models that incorporate passive digital phenotyping, active digital phenotyping, and clinical symptom interviews at different levels, allowing for a more comprehensive evaluation of sensitivity to change across time.

## Methods

### Participants

Data was collected from 50 individuals with DSM-5 diagnoses of schizophrenia or schizoaffective disorder (SZ) and 70 psychiatrically and neurologically healthy control (CN) participants. These groups did not differ on age, sex, parental education, or race; however, SZ had lower personal education and completed fewer EMA surveys than CN (see Table 3). Participants were not excluded on the basis of EMA survey completion adherence to maximize generalizability of ACL findings and because EMA surveys were not the core focus of the study. A full report of ACL and EMA adherence, feasibility, and tolerability is provided in Raugh et al.^43^. However, we have not previously reported on the validity of ACL as an objective measure of negative symptoms.

**Table 3:** Group demographic characteristics.

Variable	SZ (*n* = 50)	CN (*n* = 70)	Test Statistic
Age	38.7 (12.3)	34.7 (12.1)	*F* = 3.1
% Female	66%	71.4%	χ^2^ = 0.40
Personal education	13.2 (2.2)	15.1 (2.6)	*F* = 17.2***
Parental education	13.7 (2.9)	14.2 (2.8)	*F* = 0.86
Race			χ^2^ = 6.9
Black	32%	21.4%	
Asian-American	0%	7.1%	
Biracial	6%	4.3%	
White	58%	57.1%	
LatinX	4%	7.1%	
Other	0%	2.9%	
Survey Adherence	57%	71%	*F* = 10.3**

*CN* control, *SZ* schizophrenia.

*p < 0.05; **p < 0.01; ***p < 0.001.

SZ were recruited from local community mental health centers and online or printed advertisements. Diagnosis was made using the Structured Clinical Interview for DSM-5 (SCID)^48^. CN were recruited from the local community using printed and online advertisements. CN had no current major psychiatric diagnoses as determined via the SCID-5 (i.e., mood, anxiety, substance, eating, compulsive, traumatic, somatic), no current schizophrenia-spectrum personality disorders as determined via the SCID-PD^49^, no lifetime history of psychotic or bipolar disorders, no family history of psychosis, and were not currently prescribed any psychotropic medications. All participants reported being free from lifetime neurological disorders. Written informed consent was obtained from all participants for a protocol approved by the University of Georgia Institutional Review Board.

### Procedures

#### Phase 1: Initial laboratory visit

On the initial visit, participants provided informed consent and completed diagnostic/symptom interviews and digital phenotyping training procedures. Diagnostic/symptom interviews for SZ consisted of the SCID-5, Brief Negative Symptom Scale (BNSS)^6^, Positive and Negative Symptom Scale (PANSS)^50^, and Level of Functioning Scale (LOF)^51^. Diagnostic interviews for CN consisted of SCID-5^48^ and SCID-PD^49^. Digital phenotyping training included instructions on how to use the study phone and band, complete surveys, and manage basic troubleshooting. Participants were also instructed to keep the phone on their person. To ensure participants understood the EMA and ACL procedures, participants completed a practice EMA survey and wore the band while receiving instructions on its use and how to troubleshoot. Additionally, participants were sent home with written instructions to help with key questions or technical problems (including Bluetooth connectivity) and told to call the researchers if there were any problems. All participants were called on the first day of Phase 2 to ensure proper survey delivery and troubleshoot any issues with the band.

All participants were provided with a Blu Vivo 5R Android smartphone programmed with all surveys for Phase 2 and the Embrace band (https://www.empatica.com/embrace/). Phone functions not relevant to study procedures were blocked. Of note, consistent with prior studies^40,52,53^, we provided participants with a phone to ensure comparability of ACL data (e.g., ACL data may be calculated differently across phone operating systems or types).

#### Phase II: Digital phenotyping data collection

Surveys were preprogrammed and delivered using the mEMA application from Ilumivu (https://ilumivu.com/). In line with prior EMA studies^54–56^, surveys were scheduled to occur at eight quasi-random times between 9:00 and 21:00. A survey could occur at any time within a 90 min epoch with a minimum of 18 min and a maximum of three hours between surveys. Participants were notified of surveys by a tone and vibration (both of which could be turned off) at the scheduled time then five and ten minutes later if the survey was not completed. After 15 min, surveys became unavailable.

Surveys collected contextual variables, including current location (“Where are you?”), activity (“What are you doing?”), and social interaction (“Who are you interacting with?”) (see Supplemental Materials for response options). As participants were instructed to include activities from the 15 min preceding the survey, multiple locations, activities, and interactions could be selected. A variable delineating performance of goal-directed activities was calculated for each survey instance^36^. Specifically, we a priori identified the following survey responses as motivated or goal-directed activities: working/studying, errands/housework, exercise, shopping, or commuting/traveling. We then calculated the percentage of their EMA surveys where they were engaged in one of these goal-directed activities to achieve an index of the proportion of time participants spent in goal-directed activities. Of note, this measure of goal-directed activity encompasses but is more broad than prior papers that have used EMA to assess physical activity^57^ but largely aligns with prior EMA studies’ categorizations of effortful behavior^58^ and productive activities^59^ in severe mental illness samples.

Phone sensors were programmed to collect accelerometry values with each change in XYZ coordinate motion (every change in accelerometry being logged as a single instance), with separate values output for X, Y, and Z movement axes. The Embrace band collected accelerometry as gravitational force (g units) at a rate of 32 Hz in a range between -16g and 16 g. Embrace data was transferred to the phone via Bluetooth connection. If the connection was not available, the Embrace band could store up to 14 h of data. All data was encrypted and stored using unique identification codes on the Ilumivu or Empatica servers, separate from identifying information, until downloaded by the research team.

After 6 days of digital phenotyping data collection, participants returned the Embrace band, smartphone, and chargers and were compensated with $20 per hour for completing study interviews. Following prior methods^55,60^, participants were also compensated $1 for each EMA survey completed. Participants additionally received an $80 bonus for returning all study equipment.

### Data preparation and analysis

Accelerometry (ACL) collected by the Embrace band was aggregated into minute epochs. As recording was in g’s and XYZ angular coordinates were not recorded, continuous force of gravity is a confound in band data and cannot be removed. As such, g was converted to meters per second squared (1 g = 9.8 m/s^2^) to ensure consistency in units. Due to glitches with the smartphones, some instances had a recorded acceleration over 30 m/s^2^ (~67 mph^2^, three times the force of gravity and outside of standard human accelerometry); all such instances were excluded^61^. Axial acceleration for the phone and band were summarized as the root of the sum of the squared values.

Phone and band accelerometry data was cleaned to remove outlier values. Outliers were identified separately for each group and defined as accelerometry values greater than two times the interquartile range. Less than .1% of band accelerometry samples and 8% of phone accelerometry samples were identified as outliers and removed.

Data was analyzed at the aggregate summary level across the entire week to fall on the same timeframe as clinical rating scales. Aggregate means (ACL mean) and standard deviations (ACL SD) were calculated for each subject for phone and band data. A measure of mean “activity index” was also calculated from the band data based on an algorithm from Bai et al.^62^. This measure reflects the amount of stationary observations.

To evaluate hypothesis 1, group differences in each phone and band accelerometry measure were compared using one-way ANOVA. Bivariate correlations were used to evaluate convergent and discriminant validity for hypotheses 2 and 3. Two-tailed tests were used.

## Supplementary information

SUPPLEMENTAL MATERIALS FOR VALIDATION OF ACCELEROMETRY AS A DIGITAL PHENOTYPING MEASURE OF NEGATIVE SYMPTOMS IN SCHIZOPHRENIA

## Data Availability

All data is publicly available within the National Institute of Mental Health Data Archive (NDA) and can be accessed upon request.

## Code availability

All relevant analysis scripts are available upon request.
