# Supplementary material for: Validation of accelerometry as a digital phenotyping measure of negative symptoms in schizophrenia
Source: Schizophrenia (Heidelb). 2022 Apr 15;8(1):37. doi: 10.1038/s41537-022-00241-z (PMC9261099; doi:10.1038/s41537-022-00241-z)
Supplement: Supplementary file 1 — SUPPLEMENTAL MATERIALS FOR VALIDATION OF ACCELEROMETRY AS A DIGITAL PHENOTYPING MEASURE OF NEGATIVE SYMPTOMS IN SCHIZOPHRENIA [file 41537_2022_241_MOESM1_ESM.docx]

**SUPPLEMENTAL MATERIALS FOR**

VALIDATION OF ACCELEROMETRY AS A DIGITAL PHENOTYPING MEASURE OF NEGATIVE SYMPTOMS IN SCHIZOPHRENIA

Gregory P. Strauss, Ph.D. ^1^ *

Ian M. Raugh, B.A. ^1^

Luyu Zhang, M.A. ^1^

Lauren Luther, Ph.D.^1^

Hannah C. Chapman, B.S. ^1^

Daniel N. Allen, Ph.D. ^2^

Brian Kirkpatrick, M.D. ^3^

Alex S. Cohen, Ph.D. ^4^

1. Department of Psychology, University of Georgia
2. Department of Psychology, University of Nevada, Las Vegas
3. Department of Psychiatry and Behavioral Sciences, University of Nevada, Reno School

of Medicine

1. Department of Psychology, Louisiana State University, Baton Rouge, LA, USA

*Correspondence concerning this article should be addressed to Gregory P. Strauss, Ph.D., Email: gstrauss@uga.edu. Phone: +1-706-542-0307. Fax: +1-706-542-3275. University of Georgia, Department of Psychology, 125 Baldwin St., Athens, GA 30602.

Ecological Momentary Assessment (EMA) Questions

Context

1. Where are you? (*Multiple selection*)
   1. Home
   2. Work/ School
   3. Friend/ Family home
   4. Public place (bus, store, etc)
   5. Treatment/ Doctor’s office
2. What are you doing? (*Multiple selection*)
   1. Working/ Studying
   2. Eating/ Drinking
   3. Recreation/ Hobby
   4. Internet/ Computer use
   5. Errands/ Housework
   6. Resting
   7. Exercising
   8. Shopping
   9. TV/ Music
   10. Smoking
   11. Commuting/ Traveling
   12. Bathing/ Hygiene
   13. Pacing restlessly
   14. Socializing
   15. Nothing
3. Who are you interacting with? (*Multiple selection*)
   1. Significant other
   2. Family/ Roommates
   3. Friends
   4. Coworkers/ Classmates
   5. Doctor/ Therapist
   6. Strangers
   7. No one/ Alone

TABLE S1. The Effect of Context on Phone and Band Accelerometry

|  | **SZ**  **Mean (SD)**  **Non-Public** | **SZ**  **Mean (SD)**  **Public** | **CN**  **Mean (SD)**  **Non-Public** | **CN**  **Mean (SD)**  **Public** | **Context**  **(F, p)** | **Group**  **(F, p)** | **Group X Context**  **(F, p)** |
| --- | --- | --- | --- | --- | --- | --- | --- |
| ACLP Mean | 10.6 (.31) | 10.7 (.52) | 10.9 (.61) | 11.2 (1.2) | 3.54, .063 | 8.63, .004 | 1.15,  .29 |
| ACLP SD | 1.80 (.32) | 1.82 (.42) | 2.28 (.81) | 2.52 (.94) | 4.38, .039 | 19.84, <.001 | 3.47, .066 |
| ACLB Mean | .88 (.07) | .91 (.06) | .90 (.08) | .91 (.08) | 3.05, .088 | .24,  .63 | .55,  .46 |
| ACLB SD | .12 (.02) | .12 (.04) | .12 (.01) | .11 (.03) | .06,  .81 | .15,  .70 | 1.0,  .32 |
|  |  |  |  |  |  |  |  |
|  | **SZ**  **Mean (SD)**  **Non-Recreation** | **SZ**  **Mean (SD)**  **Recreation** | **CN**  **Mean (SD)**  **Non-Recreation** | **CN**  **Mean (SD)**  **Recreation** | **Context**  **(F, p)** | **Group**  **(F, p)** | **Group X Context**  **(F, p)** |
| ACLP Mean | 10.6 (.31) | 10.8 (.43) | 11.0 (.58) | 11.1 (.63) | 8.09, .006 | 6.99,  .01 | .23,  .63 |
| ACLP SD | 1.78 (.34) | 1.80 (.50) | 2.35 (.77) | 2.45 (1.03) | .88,  .35 | 12.7, <.001 | .42,  .52 |
| ACLB Mean | .88 (.04) | .91 (.05) | .88 (.09) | .88 (.11) | 4.13,  .05 | .05,  .83 | 2.19,  .15 |
| ACLB SD | .12 (.02) | .11 (.03) | .12 (.02) | .11 (.02) | 2.6,  .12 | .11,  .74 | .08,  .78 |
| Note. ACLP Mean = accelerometry phone mean; ACLP SD = accelerometry phone average standard deviation; ACLB Mean = accelerometry band mean; ACLB SD = accelerometry phone average standard deviation; CN = controls; SZ = schizophrenia. | | | | | | | |

TABLE S2. Correlations between Phone and Band Accelerometry

|  | Phone ACL mean | Phone ACL SD |
| --- | --- | --- |
| **Schizophrenia** |  |  |
| Band ACL Mean | .10 | .17 |
| Band ACL SD | .22 | -.24 |
| Band ACL AI | .05 | -.10 |
| **Controls** |  |  |
| Band ACL Mean | -.15 | -.10 |
| Band ACL SD | -.14 | -.08 |
| Band ACL AI | .06 | .09 |
| ACL = accelerometry; AI = accelerometry band activity index | | |
